# Pyroptotic cell death in SARS-CoV-2 infection: revealing its roles during the immunopathogenesis of COVID-19

**DOI:** 10.7150/ijbs.77561

**Published:** 2022-09-21

**Authors:** Man Wang, Wenguang Chang, Lei Zhang, Yuan Zhang

**Affiliations:** Institute for Translational Medicine, The Affiliated Hospital of Qingdao University, College of Medicine, Qingdao University, 38 Dengzhou Road, Qingdao 266021, China.

**Keywords:** SARS-CoV-2, COVID-19, cytokine storm, pyroptosis, inflammasomes, immunopathogenesis, therapeutic targets

## Abstract

The rapid dissemination of severe acute respiratory syndrome coronavirus 2 (SARS-CoV-2), the causative agent of coronavirus disease 2019 (COVID-19), remains a global public health emergency. The host immune response to SARS-CoV-2 plays a key role in COVID-19 pathogenesis. SARS-CoV-2 can induce aberrant and excessive immune responses, leading to cytokine storm syndrome, autoimmunity, lymphopenia, neutrophilia and dysfunction of monocytes and macrophages. Pyroptosis, a proinflammatory form of programmed cell death, acts as a host defense mechanism against infections. Pyroptosis deprives the replicative niche of SARS-CoV-2 by inducing the lysis of infected cells and exposing the virus to extracellular immune attack. Notably, SARS-CoV-2 has evolved sophisticated mechanisms to hijack this cell death mode for its own survival, propagation and shedding. SARS-CoV-2-encoded viral products act to modulate various key components in the pyroptosis pathways, including inflammasomes, caspases and gasdermins. SARS-CoV-2-induced pyroptosis contriubtes to the development of COVID-19-associated immunopathologies through leakage of intracellular contents, disruption of immune system homeostasis or exacerbation of inflammation. Therefore, pyroptosis has emerged as an important mechanism involved in COVID-19 immunopathogenesis. However, the entangled links between pyroptosis and SARS-CoV-2 pathogenesis lack systematic clarification. In this review, we briefly summarize the characteristics of SARS-CoV-2 and COVID-19-related immunopathologies. Moreover, we present an overview of the interplay between SARS-CoV-2 infection and pyroptosis and highlight recent research advances in the understanding of the mechanisms responsible for the implication of the pyroptosis pathways in COVID-19 pathogenesis, which will provide informative inspirations and new directions for further investigation and clinical practice. Finally, we discuss the potential value of pyroptosis as a therapeutic target in COVID-19. An in-depth discussion of the underlying mechanisms of COVID-19 pathogenesis will be conducive to the identification of potential therapeutic targets and the exploration of effective treatment measures aimed at conquering SARS-CoV-2-induced COVID-19.

## Introduction

Severe acute respiratory syndrome coronavirus 2 (SARS-CoV-2) has caused an outbreak of global viral pneumonia, named coronavirus disease 2019 (COVID-19), which poses an extraordinary threat to human health and public safety 1. After SARS-CoV and Middle East Respiratory Syndrome Coronavirus (MERS-CoV), SARS-CoV-2 is the third highly pathogenic coronavirus to spill over into human populations 2. Even with the development of vaccines, the emergence of SARS-CoV-2 variants has raised concerns regarding their enhanced infectivity and immune resistance 3. SARS-CoV-2 can cause a wide spectrum of diseases ranging from asymptomatic infection, or mild symptoms, to severe acute respiratory distress syndrome (ARDS) and death 4. Severe patients are notable with high serum levels of proinflammatory cytokines and chemokines, stressing that cytokine storm is associated with disease severity and poor clinical outcome 5. Severe COVID-19 patients experience critical tissue injuries, judged by elevated plasma levels of D-dimer and lactate dehydrogenase (LDH) 6. Leukopenia, another common symptom in severe COVID-19 cases, appears to function as an agonist of the cytokine storm 7. The release of cytokines can be the result of inflammasome activation. LDH, a hallmark of pyroptosis, is a cytosolic enzyme that is liberated to the extracellular space via ruptured membrane 8. Based on the aforementioned evidence, white cells may undergo pyroptotic cell death in severe COVID-19 cases.

A multitude of literature has manifested the implication of SARS-CoV-2 infection in regulation of inflammasome-dependent pyroptosis 9-11. Inflammasomes are momentous players in host inflammatory responses and serve as one of the first steps for induction of chronic inflammation 12. Inflammasomes are a type of cytoplasmic multiprotein complexes that typically comprise a cytosolic pattern recognition receptor (PRR; e.g., absent in melanoma 2 (AIM2)-like receptor (ALR), nucleotide-binding oligomerization domain (NOD)-like receptor (NLR) and pyrin), an adaptor protein (apoptosis-associated speck-like protein containing a caspase recruitment domain (ASC)) and an effector protein (pro-caspase-1) 13. Pyroptosis can be induced by damage-associated molecular patterns (DAMPs) and pathogen-associated molecular patterns (PAMPs) 14. Upon sensing these stressors, cytosolic PRRs initiate inflammasome formation inside the cells by recruiting ASC and pro-caspase-1. Pro-caspase-1 then undergoes proximity-induced autoproteolysis to yield enzymatically active caspase-1 within the assembled inflammasomes. Gasdermin D (GSDMD) acts as a central executor of pyroptosis and is identified as a substrate of caspase-1 15. Caspase-1 cleaves GSDMD to unleash the pore-forming N-terminal domain (GSDMD-N) and catalyzes proteolytic processing of the proforms of inflammatory cytokines (e.g., interleukin-1β (IL-1β) and IL-18) into their bioactive products 16. GSDMD-N translocates the cellular membrane where it oligomerizes and punches holes, thus facilitating the extravasation of proinflammatory mediators 17. The permeabilization of cellular membrane by GSDMD-N pores drives pyroptosis that in turn enhances the production of bioactive IL-1β and IL-18 and promotes the formation of a proinflammatory environment 18. Pyroptosis also leads to the release of DAMPs such as high-mobility group box 1 (HMGB1) and LDH, which recruit immune cells and further augment inflammation 19, 20.

Pyroptosis has emerged as a host innate immune effector mechanism against infections. Pyroptosis impedes the production of infectious viruses and facilitates the release of proinflammatory contents that recruit immune cells to combat invading pathogens 21. Conversely, excessive pyroptosis induces uncontrolled immune responses, which intensify the inflammatory damage, culminating in systemic inflammation that impels viral pathogenesis 22, 23. It is increasingly acknowledged that pyroptosis is a key contributor to COVID-19 pathogenesis. Multiple lines of evidence indicated that pyroptosis could reinforce the cytokine storm in COVID-19 patients by increasing the levels of proinflammatory cytokines 24-26. Proinflammatory factors originating from pyroptotic cells enter lungs and other organs via circulation, eventually resulting in ARDS and multiorgan injury in COVID-19 11. Therefore, inhibition of pyroptosis may be an effective approach for COVID-19 therapy. At present, some pharmaceuticals targeting upstream or downstream molecules of the pyroptosis pathway are being evaluated for their therapeutic potentials. However, considerable research efforts are still warranted to completely assess the clinical utility of these medications against COVID-19. In this review, we focus on pyroptosis-associated mechanisms involved in COVID-19 pathogenesis and discuss potential therapeutic implications of pyroptosis in COVID-19 aiming to shed light on novel countermeasures against this devastating disease. A more sophisticated comprehension of the roles of pyroptosis in SARS-CoV-2 immunopathogenesis is instrumental in the development of effective therapeutic approaches capable to repress viral replication and curb the hyper-inflammation in COVID-19 patients.

## The characteristics of SARS-CoV-2

**Figure 1:**
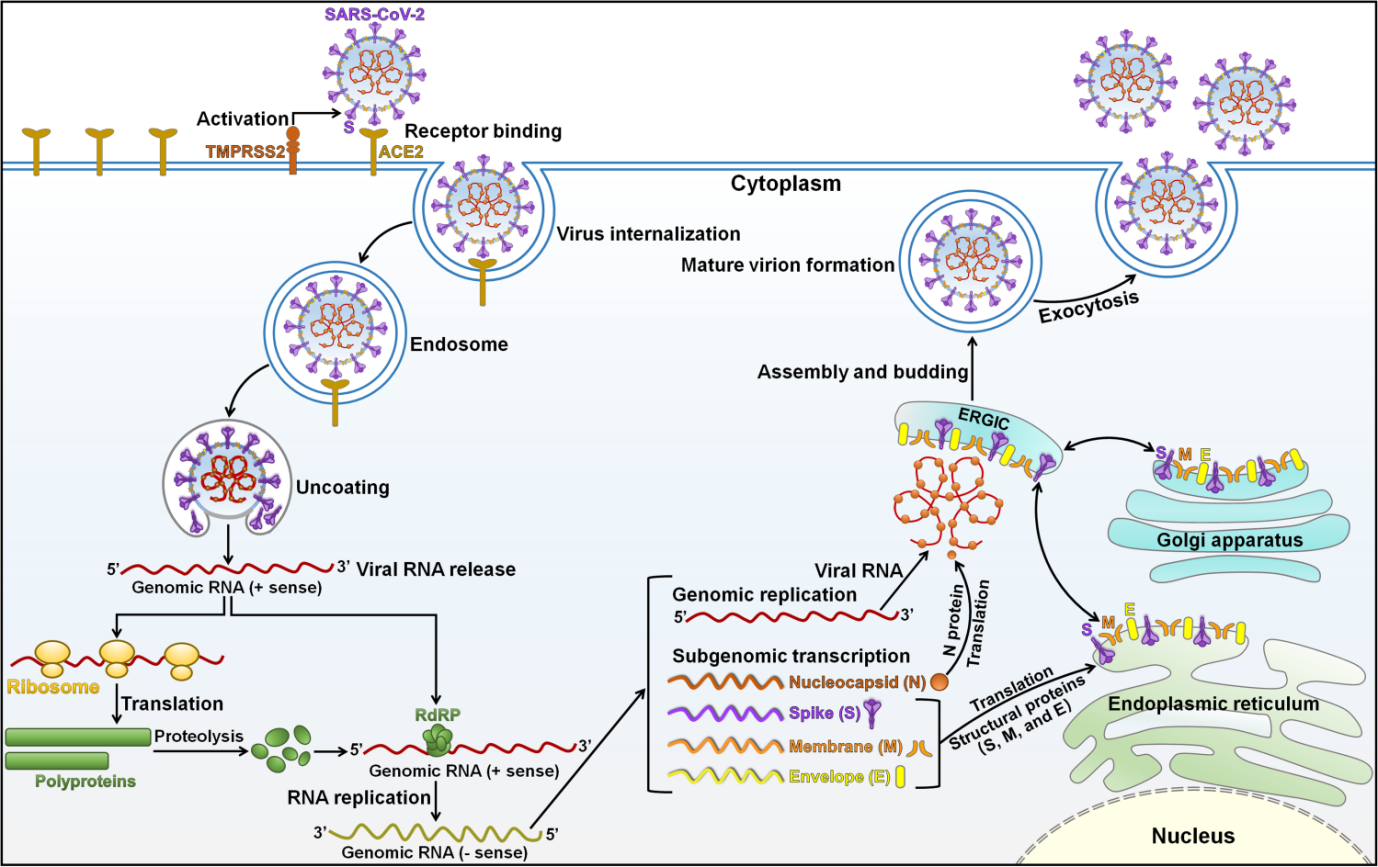
** Schematic representation of the SARS-CoV-2 life cycle**. The binding of SARS-CoV-2 spike (S) protein to the ACE2 receptor on the host cell surface leads to the fusion of viral and cellular membrane. The cellular surface serine protease TMPRSS2 is required for the priming of viral S protein. SARS-CoV-2 enters the host cell through the endosomal pathway, followed by the release of nucleocapsid into the cytoplasm. The virus then dissolves its protein shells and releases its genome inside the cell. The viral RNA attaches to the cell translation machinery to synthesize two large polyproteins (pp1a and pp1ab). These polyproteins are broken down into smaller NSPs by host and viral proteases. Among them, RdRp is the core catalytic subunit that drives the replication and amplification of viral RNA genome. The resultant negative-sense RNA acts as a template to produce genomic RNA and a collection of subgenomic RNAs (sgRNAs) that encode viral structural and accessory proteins. The viral RNA and N protein are synthesized in the cytoplasm. Viral S, M and E proteins are translated from sgRNAs by the ribosomes present in the endoplasmic reticulum (ER) and are subsequently embedded in an intermediate compartment of ER with Golgi (ERGIC). The viral genome is encapsulated by N proteins and assembled with the structural proteins in ERGIC. The mature virion is formed by budding from the lumen of ERGIC. The progeny virus is then liberated into the extracellular environment via exocytosis. SARS-CoV-2, severe acute respiratory syndrome coronavirus 2; TMPRSS2, transmembrane protease serine 2; ACE2, angiotensin-converting enzyme 2; RdRp, RNA-dependent RNA polymerase; ERGIC, endoplasmic reticulum-Golgi intermediate compartment; N, nucleocapsid; S, spike; M, membrane; E, envelope.

SARS-CoV-2 is a member of the genus* Betacoronavirus* in the family *Coronaviridae* 27. SARS-CoV-2 is an enveloped, non-segmented positive-sense, single-stranded RNA virus with a genome of approximately 30 kilobases (kb) 28. SARS-CoV-2 contains four structural proteins (spike (S), membrane (M), envelope (E), and nucleocapsid (N)) and sixteen Non-Structural Proteins (NSP1-16) 29. The viral genome is incorporated by N proteins, which are embedded inside phospholipid bilayers and are covered by S proteins 28. M and E proteins reside among S proteins in the viral envelope. The four structural proteins are instrumental in viral infection and virion production. SARS-CoV-2 infection is initiated by using its attachment protein S (Figure 1). The glycoprotein S forms homotrimers protruding from the viral surface that mediate direct contact with the cellular receptor angiotensin-converting enzyme 2 (ACE2) 30. After binding to the host receptor, S protein undergoes proteolytic cleavage by the cell surface transmembrane protease serine 2 (TMPRSS2) and endolysosomal cathepsin L, contributing to the generation of two subunits S1 and S2 31. The S1 subunit attaches to the ACE2 receptor on the host cell via its receptor-binding domain (RBD), and the S2 subunit has the ability to promote the fusion of viral and host lipid bilayers. It is followed by various steps including internalization of the virus, disassembly of viral capsid, and delivery of its genome to the host cell cytoplasm 32, 33. The viral RNA genome is translated into essential enzymes for RNA synthesis (NSP12, RNA-dependent RNA polymerase), proofreading (NSP14) and capping (NSP14-16) 34. The replicase components re-organize the endoplasmic reticulum (ER) into double-membrane vesicles (DMVs), where the viral genome replicates to create full-length copies 35. The resultant negative-stranded RNA intermediate acts as the template for synthesis of a full-length genomic RNA and a nested set of subgenomic RNAs (sgRNAs) through a discontinuous transcription mechanism 36. The synthesized sgRNAs released from DMVs are translated into viral structural and accessory proteins that are embedded into the ER-Golgi intermediate compartment (ERGIC) for the assembly and release of new virions. Specifically, viral particle assembly initiates with the encapsidation of genomic RNAs with N proteins, forming nucleocapsid structures that bud into the lumen of secretory vesicular compartments 37. Newly produced virions are released from the host cell via exocytosis. SARS-CoV-2 structural proteins play a vital role in its life cycle. The protein N can interact with encapsidated genome to assist in viral genome packaging. The protein M functions to prompt virion assembly and comprises three transmembrane domains, which facilitate membrane curvature that can encompass the nucleocapsid 38. The protein E is engaged in viral pathogenesis and plays a critical role in virion assembly and release 39.

COVID-19 is a highly contagious disease caused by SARS-CoV-2 40. SARS-CoV-2 is able to infect humans and other mammals, and it leads to respiratory, musculoskeletal, neurologic, and gastrointestinal system diseases. Its main clinical manifestations are chills, cough, sore throat, chest pain, hemoptysis, shortness of breath, myalgia, ageusia, anosmia, confusion, headache, diarrhea, nausea and vomiting 41. The most common complications include ARDS, pneumonia, cardiac injury, liver dysfunction, renal disease, thrombosis and septic shock, which result in severe disease and death in COVID-19 patients 42. The average incubation period for COVID-19 is 5-6 days 43. This disease is mild in nearly 90% of infected individuals, but it may progress from pneumonia or systemic inflammation to multiorgan dysfunction in a minority of cases 44. Risk factors for severe COVID-19 include preexisting complications (e.g., cardiovascular disease, diabetes and hypertension), immunodeficiency, and older age. Obesity is a chronic low-grade inflammatory disease, which is characterized by enhanced release of proinflammatory cytokines from the adipose tissue and the infiltration of leukocytes (e.g., macrophages) into the adipose tissue 45. Particularly, obesity represents a clinically significant risk factor for poor outcomes in COVID-19 46. Adipocytes express high levels of ACE2 and other entry receptors (e.g., FURIN, NRP1 and TFRC) for SARS-CoV-2 47. Thus, adipocytes are susceptible to SARS-CoV-2 infection. The adipose tissue acts as a reservoir of SARS-CoV-2 spread/shedding and cytokine amplification 48. Furthermore, excessive adiposity exacerbates SARS-CoV-2-induced cytokine storm in COVID-19 patients. Recently, a large population-based cohort study indicated the high levels of vaccine effectiveness against severe COVID-19 disease in people with overweight and obesity 49. The time from SARS-CoV-2 infection to death ranges from 6 to 41 days. SARS-CoV-2 is predominantly transmitted through respiratory droplets during close or unprotected direct contact between infected and uninfected individuals 50. Other modes of transmission have also been proposed, including aerosol, the fecal-oral route and indirect contact. Notably, there is valid evidence for asymptomatic spread of SARS-CoV-2. In addition to clinically approved vaccines, personal preventive manners such as social distancing, avoidance of social gatherings, keeping hand-hygiene, wearing masks, frequent testing and contact tracing can control the transmission of SARS-CoV-2 among human populations 51.

The outbreak of COVID-19 has pushed the world to the center of a new pandemic. Considerable research effort has been invested in seeking means to restrict SARS-CoV-2 spread and to treat COVID-19 through vaccines and therapies. Increasing knowledge of the pathogenicity, epidemiology and clinical features of SARS-CoV-2, along with organization and function of viral genome, will provide the basis for the development of new preventive and treatment approaches against COVID-19. Although experimental studies have made a breakthrough, there are significant gaps in the available evidence on SARS-CoV-2 biology. Like other RNA viruses, SARS-CoV-2 is prone to develop mutations, hence facilitating rapid virus evolution. Some variants such as B.1.351 (Beta), B.1.617.2 (Delta) and B.1.1.529 (Omicron) harbor mutations that make them more insusceptible to host immunity 52-54. These mutant strains are characterized by high transmissibility and infectivity, which can be partially attributed to the key mutations spanning viral S protein. Given that distinct viral strains of SARS-CoV-2 have been implicated in this epidemic, the genuine evolution origin of this virus awaits further survey. Subsequent genomic supervision of SARS-CoV-2 in new infection cases will help to recognize the mutations that may lead to phenotypic alterations of this virus. Continued studies are required to decipher how the variants evade from either infection-induced or vaccine-mediated immune responses. It is likely that accessory proteins of SARS-CoV-2 play a key role in viral replication. The exact function of viral accessory proteins is yet to be defined due to their limited homology to known proteins or to those of other coronaviruses. Additional work is necessary to clarify the complicated and dynamic interactions between SARS-COV-2 and the host. Moreover, key host factors associated with SARS-CoV-2 infection remain to be systematically identified and characterized. It is intriguing how viral or host factors affect the progression and severity of COVID-19. Overall, fighting the COVID-19 epidemic is challenging and a long-term job that requires collective global efforts.

## The immunopathology of COVID-19

**Figure 2:**
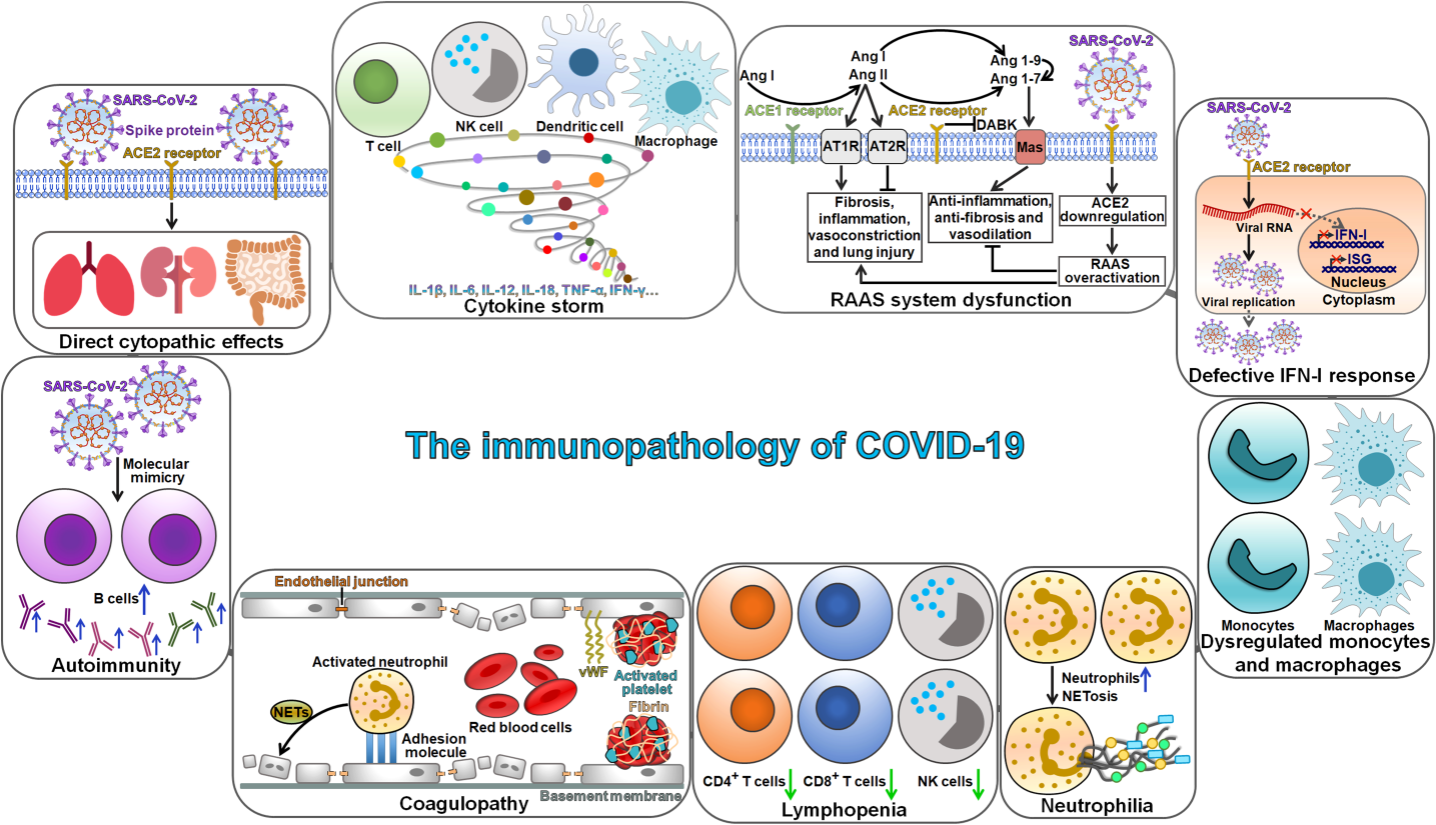
** The immunopathology of COVID-19**. SARS-CoV-2 infection can directly cause damage to various tissues, such as lungs, kidneys and intestine. Multiple key immunopathological characteristics may be associated with COVID-19 pathogenesis, including cytokine storm, RAAS system dysfunction, autoimmunity, coagulopathy, lymphopenia, neutrophilia, dysregulated monocytes and macrophages, and defective IFN-I response. SARS-CoV-2, severe acute respiratory syndrome coronavirus 2; ACE2, angiotensin-converting enzyme 2; NK cell, natural killer cell; IL-1β, interleukin-1β; IL-6, interleukin-6; IL-12, interleukin-12; IL-18, interleukin-18; TNF-α, tumor necrosis factor-α; IFN-γ, interferon-γ; Ang I, Angiotensin I; Ang II, Angiotensin II; ACE1, angiotensin-converting enzyme 1; AT1R, angiotensin type 1 receptor; AT2R, angiotensin type 2 receptor; DABK, des-Arg^9^-bradykinin; Ang 1-9, angiotensin 1-9; Ang 1-7, angiotensin 1-7; RAAS, renin-angiotensin-aldosterone system; IFN-I, interferon-I; ISG, interferon-stimulated gene; NETosis, neutrophil extracellular trap formation; vWF, von-Willebrand factor.

The mechanism of SARS-CoV-2 pathogenesis is not well understood. The invasion of SARS-CoV-2 triggers cytokine storm syndrome and severe immune responses, which are thought to promote disease pathogenesis and result in severe complications and pulmonary deterioration 55. The immunopathogenesis of SARS-CoV-2 has become a research hotspot in this field. COVID-19 progression is deemed as a consequence of complicated interplays among various pathophysiological mechanisms, such as direct cytopathic effects of SARS-CoV-2, dysregulation of the renin-angiotensin-aldosterone system (RAAS) and activation of des-Arg^9^-bradykinin (DABK) mediated by SARS-CoV-2-induced ACE2 inhibition, cytokine storm syndrome, autoimmunity and coagulopathy (Figure 2). Moreover, lymphopenia, neutrophilia and dysfunction of monocytes and macrophages are also key characteristics of immunopathology in COVID-19 patients.

## SARS-CoV-2-induced cytopathic effects through ACE2

The pathogenesis of COVID-19 may be divided into two phases 56. The first phase (the viral phase) is characterized by the production of new viral particles contributing to direct cytopathic effects. The extent of SARS-CoV-2-induced damage determines the development of the second phase. In the later phase, effector immune cells are recruited to the site of infection leading to local and systemic inflammatory responses that may persist even after viral clearance. ACE2 is highly expressed in most human organs, including duodenum, heart, intestine, kidneys and lungs 57. It is not surprising that SARS-CoV-2 infection can cause systemic symptoms. SARS-CoV-2 enters the human body primarily through ACE2-expressing nasal epithelial cells 58. It then descends the respiratory tract and invades the alveolar region of lungs. Blood vessels are also critical target tissues that may act as a viral spread route within the human body 59. SARS-CoV-2 infection and replication can induce cytopathic effects and lead to decreased viability in host cells 60. COVID-19 clinical manifestations may be partially attributable to the targeting of ACE2-expressing tissues by SARS-CoV-2. Pulmonary alveolar cells possess high levels of ACE2. SARS-CoV-2 infection in alveolar epithelial cells triggers destruction of vascular integrity, such as increased vascular permeability and leakage, which leads to hypoxic respiratory failure, disseminated intravascular coagulation (DIC), pulmonary ischaemia and oedema, and progressive lung damage 61. Gustatory and olfactory abnormalities in COVID-19 cases may result from the invasion of SARS-CoV-2 into olfactory cavity 62. It is perceived that SARS-CoV-2 can break through the blood-brain barrier and reach the central nervous system (CNS), thus contributing to neurologic manifestations, such as headache, dizziness and encephalopathy 63. SARS-CoV-2 exerts cytopathic effects on ACE2-expressing podocytes and proximal straight tubule cells within the kidneys, hence inducing acute renal injury in COVID-19 patients 64. SARS-CoV-2 infection can also cause gastrointestinal symptoms, such as inflammation, malabsorption and diarrhea. The pathogenesis of extra-pulmonary symptoms may be attributed to a diversity of factors, such as SARS-CoV-2-mediated damage to neurons and tissue cells, excessive cytokine secretion, antibody-induced tissue dysfunction, or vascular injury. The pathophysiological mechanisms underpinning COVID-19 development remain to be further investigated. ACE2 has emerged as an important inducer of pyroptosis during SARS-CoV-2 infection 65, 66. ACE2-mediated SARS-CoV-2 infection activated NLR family, pyrin domain-containing protein 3 (NLRP3) inflammasome and drove pyroptosis in human macrophages 67. Moreover, proinflammatory cytokines released from pyroptotic cells shaped the hyper-inflammatory macrophage response and impelled immunopathology associated with chronic SARS-CoV-2 infection. The interaction between viral S protein and ACE2 receptor was proposed to cause the hyper-activation of NLRP3 inflammasome which led to the elimination of infected cells via pyroptosis 66, 68. Therefore, ACE2 may contribute to COVID-19 immunopathogenesis through induction of pyroptosis. The involvement of ACE2-mediated pyroptosis in direct cytopathic effects caused by SARS-CoV-2 infection warrants further study.

## SARS-CoV-2-associated cytokine storm

The immune system constitutes one of the most crucial defense mechanisms against viral infections 69. After entering the lungs through respiration, SARS-CoV-2 activates immune cells, stimulates cytokine secretion, and instigates other antiviral mechanisms 70. Nevertheless, exaggerated immune responses may result in immune damage to the human body 71. The pathogenicity of COVID-19 can result from overactivation of host immune response or abnormal immune reaction in infected individuals. Cytokine storm syndrome is a series of excessive immune responses that drive immune system exhaustion, eventually leading to organ damage and fatal respiratory distress 72. The cytokine storm is characterized by rapid production of massive proinflammatory cytokines, which direct immune cells to the sites of inflammation. The overactivation of immune cells can lead to the exponential growth of inflammation and organ failure.

The cytokine storm is implicated in the pathogenesis of COVID-19 and has a close relationship with disease deterioration in COVID-19 patients 73. SARS-CoV-2 infection elicits both innate and adaptive immune responses. Once activation by SARS-CoV-2, multiple cytokines and mediators released by CD4^+^ T cells are able to stimulate B cells and cytotoxic T cells. The activated B cells produce virus-specific antibodies, and the stimulated CD8^+^ T cells mediate the clearance of virus-infected cells. However, SARS-CoV-2 has managed to evade host immune attack and constantly produces viral nucleic acids that stimulate immune responses. Dysregulated host immune responses caused by SARS-CoV-2 give rise to extremely increased levels of chemokines (e.g., C-C motif chemokine ligands (CCLs)), proinflammatory cytokines (e.g., IL-1β, IL-6, IL-12, tumor necrosis factor (TNF) and interferon-γ (IFN-γ)) and free radicals, culminating in the development of cytokine storm syndrome 74. IL-6 is a key factor in cytokine storm. IL-6 can directly trigger the inflammation mediator nuclear factor-κB (NF-κB) signaling cascade via PPRs 75. Moreover, IL-6 also activates signal transducer and activator of transcription 3 (STAT3) in non-immune cells by forming the IL-6/IL-6 receptor (IL-6R) complex 76. NF-κB and STAT3 exert a synergistic role in promotion of IL-6 transcription, forming a positive feedback for the IL-6 signaling and thus amplifying inflammation. Proinflammatory cytokines and chemokines recruit monocytes and neutrophils to the lung tissues, fostering extensive infiltration of inflammatory cells that aggrandizes the lung injury 71. Cytokine storm syndrome can strengthen the apoptosis of epithelial and endothelial cells in the lungs, resulting in vascular leakage and ultimately ARDS 77. The main changes in the lung tissues of COVID-19 patients include alveolar edema and proteinaceous exudates, apparent desquamation of pneumocytes and hyaline membrane formation, diffusion alveolar damage and thickening of alveolar walls, all of which are clinical manifestations of ARDS 78. ARDS, an immune-associated clinical feature of SARS-CoV-2 infections, is the leading cause of disease severity and mortality in COVID-19 patients 79.

## SARS-CoV-2-mediated RAAS system dysfunction

ACE plays an important role in the RAAS system. There are two distinct isoforms of ACE, ACE1 and ACE2. ACE1 is abundantly expressed in vasculature and renal proximal tubules 80, and ACE2 is mainly distributed in the heart, lungs and kidneys 81. ACE2 converts the active bradykinin metabolite DABK into a biologically inactive form 82. Conversely, downregulation of ACE2 inhibits DABK inactivation. DABK functions as a ligand for bradykinin 1 receptor (B1R), while bradykinin is an agonist for bradykinin 2 receptor (B2R). B1R and B2R stimulation through their ligands leads to enhanced vascular permeability and neutrophil attraction, thereby facilitating inflammation. Angiotensin II (Ang II) is a main physiological product of the RAAS cascade. ACE1 is responsible for the transformation of Ang I into Ang II. ACE2 is a homolog of ACE1 and converts Ang I and Ang II to bioactive products of the RAAS system, namely Ang 1-9 and Ang 1-7 83. Ang 1-9 and Ang 1-7 are able to facilitate vasodilation and restrain inflammatory responses. The actions of Ang II result from motivating its specific receptors, the angiotensin type 1 receptor (AT1R) and type 2 receptor (AT2R) 84. AT1R activation leads to atherogenesis, inflammation and vasoconstriction 85, while stimulation of the Ang II/AT2R signaling gives rise to decreased platelet aggregation, enhancement of insulin action and vasodilation. Excessive Ang II accumulation induces cytokine-induced and inflammatory organ damage, and pulmonary vasoconstriction. The proinflammatory cascade and elevated vascular permeability induced by ATR overactivation in the lungs contributes to acute lung injury, ARDS and even death 86, 87. The interaction between SARS-CoV-2 and ACE2 restricts the function of ACE2 by reducing its surface expression, leading to RAAS dysfunction, Ang II accumulation and elevated levels of DABK. Activation of RAAS leads to elevated levels of Ang II, which is a pivotal initiator of NLRP3 inflammasome 88. Ang II is proposed to hyper-activate NLRP3 inflammasome in SARS-CoV-2-infected cells after binding to AT1R, contributing to pyroptosis and discharge of proinflammatory cytokines. RAAS system dysfunction may instigate the cytokine storm in COVID-19. Accordingly, SARS-CoV-2-mediated inhibition of ACE2 may result in increased availability of diverse molecules that are associated with hyper-inflammatory response, hence leading to COVID-19 progression 89.

## SARS-CoV-2-induced autoimmunity and coagulopathy

Autoimmunity may be formed by a wide range of factors through establishment of a hyper-stimulated state of the immune system 90. Viral infection constitutes a significant contributor to the onset of autoimmune diseases. It has been well established that SARS-CoV-2 induces an overstimulated state of host immune system. SARS-CoV-2 infection is linked with a sharp increase in the levels of proinflammatory cytokines and alternations in circulating lymphocyte populations 91. In some cases, SARS-CoV-2 infection induces aggressive immune responses, leading to severe inflammation and damage to crucial organs. Exaggerated activation of host immune system is a key determinant of the severity of illness and mortality in COVID-19 patients. The creation of autoantibodies is a vital characteristic of autoimmune diseases. B cells confer protection against SARS-CoV-2 infection by generating neutralizing antibodies to restrict viral entry 92. It should be noted that B cell-synthesized neutralizing antibodies are not always advantageous for the host, partially depending on the viral component it targets. Molecular mimicry by invading pathogens is one of the underlying mechanisms responsible for the production of autoantibodies. Viral infection can interrupt immune tolerance by exposure of antigen epitopes that elicit cross-reactive antibodies. Multiple epitopes from SARS-CoV-2 have been found to show cross-reactivity with host autoantigens. For instance, a unique S1/S2 cleavage site in SARS-CoV-2 completely mimicked a FURIN-cleavable peptide on the human epithelial sodium channel α-subunit (ENaC-α), which exerted a pivotal role in the homeostasis of airway surface lipid 93. Mimicry between SARS-CoV-2 and three proteins (AIFM, DAB1 and SURF1) from the human brainstem pre-Bötzinger complex (preBötC) might result in respiratory failure in COVID-19 94. Particularly, SARS-CoV-2 can trigger autoimmune responses via molecular mimicry. Peptides embedded in immunoreactive epitopes of SARS-CoV-2 showed homology with heat shock protein 60 (HSP60) and HSP90, which were connected to Guillain-Barré syndrome and other autoimmune disorders 95. Several human pulmonary-arterial peptides mimicked by SARS-CoV-2 were able to associate with HLA-B*35:01, HLA-B*40:01 and HLA-B*40:02, which played an intricate role in mediating immune responses 96. Autoimmune disorders are defined as a heterogeneous group of diseases in which the immune system attacks the body's own tissues and organs 97. Pyroptosis, a key component of innate immunity, plays a pivotal role in the occurrence and progression of autoimmune disorders. Excessive pyroptosis interrupts immune system homeostasis and thus initiates autoimmunity 98. Uncontrolled release of inflammatory mediators from pyroptotic cells results in a hyper-activated immune system, which further exacerbates autoimmune diseases 99. Moreover, proinflammatory cytokines represent a functional bridge between innate and adaptive immunity by attracting and activating immune cells (e.g., T cells) 100. Aberrant activation of adaptive immunity leads to the impairment of autoimmune system and destruction of immune tolerance to normal tissues. Under such circumstances, autoantibodies and autoreactive immune cells erroneously attack specific tissues in the body, culminating in autoimmune diseases 101. It is reasonable to assume that there is a causal relationship between SARS-CoV-2-induced pyroptosis and the pathogenesis of autoimmune diseases. Further research efforts are required to corroborate this hypothesis.

Reportedly, COVID-19 patients tended to have an increased incidence of coagulation disorders 102. Coagulopathy is a life-threatening complication of SARS-CoV-2 infection. Pathophysiology of COVID-19-associated coagulopathy primarily depends on a multitude of complicated interplays between excessive secretion of proinflammatory factors, platelet overactivation, and endothelial dysfunction 103. For example, the proinflammatory cytokine tissue factor (TF) serves as a principal initiator of the extrinsic coagulation cascade and contributes to a hyper-coagulable state 104. Upon stimulation by inflammatory mediators, activated neutrophil-secreted neutrophil extracellular traps (NETs) could induce platelet activation and mediate endothelial dysfunction, ultimately causing coagulation diseases in COVID-19 patient 105. Other than aforementioned abnormalities, alternative pathogenic mechanisms, such as overproduction of von-Willebrand factor (vWF) from impaired endothelium, stimulation of toll-like receptors (TLRs) and complement activation, also participate in COVID-19-associated coagulopathy. Specifically, damaged endothelial cells liberate ultra-large vWF multimers which in turn promote platelet activation and adhesion 106. Overexpressed TLR9 was revealed to foster platelet hyper-activation and thrombosis by motivating the protein kinase B (Akt) and IL-1R-associated kinase 1 (IRAK1) pathways 107. The complement system is a portion of the innate immune system and consists of many components including C3a, C5a and C5b 108. The interaction between complement proteins and coagulation gives rise to a hyper-coagulable state, inflammation and thrombotic microangiopathy in COVID-19 103. Currently, the detailed mechanism of coagulation is still obscure, it may be a result of virus-induced injury to the vasculature or severe inflammatory responses, which change the vascular endothelium and activate macrophages, monocytes and platelets. These events enhance the discharge of TF, vWF and factor VIII, eventually promoting the formation of thrombin and fibrin clot. It is now established that pyroptosis represents an important mechanism linking inflammation to coagulation. Inflammasome activation and subsequent pyroptosis led to the development of DIC and thrombosis throughout the vasculature 109-111. DIC is a catastrophic syndrome characterized by widespread activation of blood coagulation, which results in hemorrhage, thrombotic obstruction of microvessels and organ failure 112. Moreover, TF released from pyroptotic macrophages and monocytes could induce systemic coagulation and extensive thrombosis in tissues, contributing to multiorgan damage and host lethality 109, 113. The lytic cell death may be implicated in COVID-19 coagulopathies. SARS-CoV-2-induced pyroptosis in endothelial cells contributes to the extravasation of intracellular contents such as TF, plasminogen-activating inhibitors and procoagulant factors 114. These factors likely enter neighboring endothelial cells and the blood stream, leading to local and systemic coagulopathies in COVID-19 patients. However, the role of pyroptosis in the initiation and progression of coagulopathy during COVID-19 remains to be better defined. The precise mechanisms underlying COVID-19-associated coagulopathy are worthy of in-depth investigation.

## Other key immunopathological features of COVID-19

Lymphopenia, also known as lymphocytopenia, refers to a dramatic decrease in the number of lymphocytes in the blood 115. Lymphopenia can be further categorized based on the type of lymphocytes (B cells, natural killer (NK) cells and T cells) that are depleted 116. Increasing evidence has manifested the correlation between lymphopenia and disease severity in COVID-19 patients 117. Particularly, lymphopenia is commonly found in severe cases. A previous study manifested that COVID-19 patients with severe illness exhibited a significant decrease in CD4^+^ and CD8^+^ T cells 118. Moreover, the levels of these lymphocyte subsets reflected the disease deterioration and were related to clinical outcomes. Lymphocyte counts reverted to normal range upon recovery 119. Therefore, the levels of CD4^+^ and CD8^+^ T cells might be good biomarkers of COVID-19. The serum levels of proinflammatory cytokines (IL-6 and TNF-α) were intimately linked with lymphopenia 7. Thus, the inflammatory cytokine storm may be conducive to the development of lymphopenia. Furthermore, high serum levels of IL-6 might have a relationship with defective cytotoxic activity of both T cells and NK cells 120. On the contrary, IL-6 blockade could increment the counts of circulating lymphocytes. Targeting proinflammatory cytokines may restore host antiviral mechanisms. Further studies are required to uncover the mechanisms by which the cytokine storm affect the number and activity of lymphocytes. SARS-CoV-2 infection seemed to hinder T cell expansion marked by downregulation of genes related to T cell activation and function 121. Nevertheless, the direct and indirect effects of SARS-CoV-2 on lymphopenia development in COVID-19 need particular attention. Collectively, the underlying mechanisms responsible for lymphopenia in COVID-19 remain largely unknown, and concerted research efforts are demanded to expand the boundaries of our knowledge about SARS-CoV-2-triggered lymphopenia.

Neutrophils are the most eminent innate cells in viral defense. Neutrophilia is a condition defined by an increased count of neutrophils in the peripheral blood 122. A large number of neutrophils swarm to the infection site, which definitely results in extensive pathology 123. Excessive counts of peripheral neutrophils were proven to correlate with illness severity and poor outcome in COVID-19 patients 124. Neutrophils were found to infiltrate pulmonary capillaries, and the expression levels of neutrophil-related chemokines were elevated in lung epithelial cells in COVID-19 patients 125. These studies suggested the contribution of neutrophilia to COVID-19 pathology. The formation of NETs is an important mechanism of neutrophil action. NETs are neutrophil-produced extracellular fibers comprising of chromosomal DNAs, histones and granule proteins, which can ensnare and kill extracellular pathogens 105. The process of NET formation, called NETosis, was revealed to correlate with disease severity in COVID-19 patients 126. NETosis may facilitate fibrosis or constitute a chain reaction with coagulation factors, complement and platelets culminating in immunothrombosis, a process featured by blood clotting and endothelial dysfunction 127. NETs mediated thrombotic complications in COVID-19 patients, involving complement (C3), TF and platelet in the blood 128. NET-relevant thrombotic events were also regarded as a central driver of lung pathology, organ failure and even death in COVID-19 patients 129. Reportedly, IL-6 had the ability to induce neutrophilia and to regulate neutrophil trafficking under inflammation 130. IL-6-mediated activation of complements played an essential role in NET production 131. The participation of IL-6 in regulation of neutrophilia and NETosis remains to be determined. The release of DAMPs from infected cells, the cytokine storm and viral infection-induced cell death are likely to cause NETosis in COVID-19. Substantial research efforts are warranted to unravel the regulatory mechanisms of neutrophilia and NETosis in COVID-19.

Monocytes and macrophages are important innate immune cells with prominent defensive activities against invading microbes 132. They respond to microbial antigens by generating proinflammatory mediators to eliminate microbes and repair tissue damage 133. However, monocytes and macrophages may present deleterious actions on the host during SARS-CoV-2 infection. Mechanistically, SARS-CoV-2 can infect monocytes and macrophages to block their defensive functions 10. The resultant dysfunction of these immune cells contributes to severe organ damage by magnifying the acute inflammation, fostering the cytokine storm, interacting with adaptive immune cells and promoting fibrotic complications 134. Accordingly, dysregulated monocytes and macrophages aggravate the disease to a very high extent and lead to ARDS, which is a common cause of death in COVID-19 patients 135. It is still intriguing how SARS-CoV-2 affect the activity of monocytes and macrophages. Recent studies showed that SARS-CoV-2 infection was capable of triggering pyroptosis in both monocytes and macrophages, providing a potential mechanistic explanation for the dysfunction of these cells 136, 137. The mechanisms associated with the immunopathological enhancement of COVID-19 by monocytes and macrophages require further researches.

Pathogen infection can stimulate host innate immune system that recognizes pathogens and induces the release of proinflammatory cytokines that recruit immune cells to the infection site to trigger an inflammatory response 138. It is followed by the activation of adaptive immunity, manifested by T cell-mediated removal of pathogen-infected cells and B cell-secreted pathogen-specific antibodies. Remarkably, infection-induced immunity not only plays a critical role in viral clearance, but also impairs host tissues. Uncontrolled production of proinflammatory cytokines and chemokines (e.g., IL-1β, IL-6 and IL-8) attracts unessential influx of immune cells to the infected sites. These immune cells may cause inflammatory damage to the lungs and other tissues by releasing toxic granules. Since excessive accumulation of activated immune cells is observed in COVID-19 patients, organ failure is more likely to result from a hyper-activated immune system or vascular damage, instead of direct viral injury. The genuine contribution of viral loads to disease outcome in COVID-19 has yet to be characterized, and thus further study is needed. Importantly, the host may be incapable of opposing SARS-CoV-2-induced inflammatory responses due to dysregulation of antiviral immune system. For instance, defective type I interferon (IFN-I) immunity was observed in COVID-19 patients, as evidenced by the production of autoantibodies against IFN-α and IFN-ω, which led to delayed viral clearance 139. Moreover, SARS-CoV-2 infection resulted in a marked decline in the number of dendritic cells (DCs) and T cells, thereby weakening T cell-mediated antiviral immunity 140. Defective type 1 (antiviral) immunity and elevated type 2 (anti-helminths) immune responses had been connected with disease severity in COVID-19, hinting that an imbalanced adaptive immunity might act as a driver of COVID-19 development 141. In short, the immunopathology of COVID-19 is multifactorial, involving impaired induction of IFN, exuberant inflammatory responses and delayed adaptive immunity. The profound impacts of SARS-CoV-2 infection on host immune system are still worthy of in-depth study. CD8^+^ T cells may play dual roles in COVID-19 progression 142, so the heterogeneity of virus-specific T cells should be further dissected. A universal understanding of immunopathogenic alterations induced by SARS-CoV-2 infection will facilitate the development of better therapeutic solutions. Prevention of SARS-CoV-2-induced overinflammation or restoration of host immunity may hold great promise for the treatment of COVID-19. The exploitation of immune cell therapy for the removal of virus-infected cells could induce excessive inflammation, so this treatment must be applied in conjunction with other therapeutic options.

## Pyroptotic cell death during SARS-CoV-2 infection

**Figure 3:**
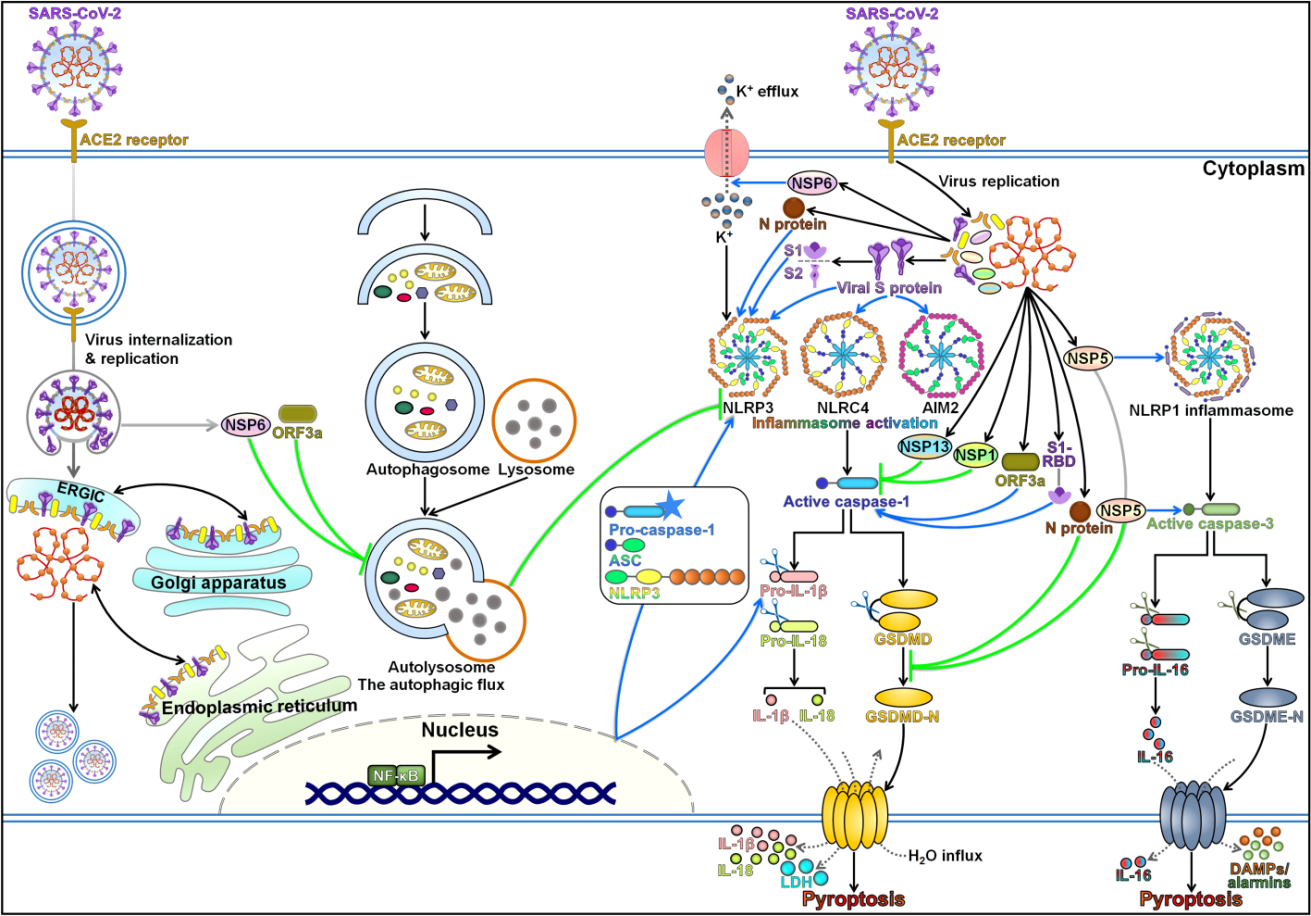
** Regulatory mechanisms of pyroptotic cell death during SARS-CoV-2 infection.** Pyroptosis has been closely linked with SARS-CoV-2 pathogenesis. SARS-CoV-2 infection coordinates the activation of the pyroptosis pathways through various mechanisms. Once SARS-CoV-2 enters the host cell, the viral genome is released and starts to replicate and manufacture a series of proteins to assemble new virions. Viral NSP6 and ORF3a trigger NLRP3 inflammasome-dependent pyroptosis by impeding the autophagic flux. NSP6 promotes the assembly of NLRP3 inflammasome by inducing K^+^ efflux. SARS-CoV-2 infection may stimulate the NF-κB signaling pathway, hence driving NLRP3 inflammasome-mediated pyroptosis. Moreover, viral attachment protein S and its S1 subunit facilitate the activation of NLRP3 inflammasome and induce GSDMD-executed pyroptosis in infected cells. These events cause the extravasation of proinflammatory mediators (e.g., IL-1β and IL-18), LDH release and eventually prompt robust immune reactions. S protein also has the ability to motivate NLRC4 and AIM2 inflammasomes, suggesting that it may affect diverse pyroptosis-related signaling cascades. Viral NSP1 and NSP13 inhibit caspase-1 activation, while ORF3a and S1-RBD exert opposite effects. N protein supports the formation of NLRP3 inflammasome, while it antagonizes pyroptotic cell death in host cell by inactivating the pyroptosis executioner GSDMD. Likewise, NSP5 can prevent GSDMD-mediated pyroptosis. Instead, NSP5 actuates the NLRP1/caspase-3/GSDME pathway. It is followed by the formation of GSDME-N pores on the cellular membrane, contributing to lytic cell death and the secretion of proinflammatory cytokines, DAMPs and alarmins. SARS-CoV-2, severe acute respiratory syndrome coronavirus 2; ACE2, angiotensin-converting enzyme 2; ERGIC, endoplasmic reticulum-Golgi intermediate compartment; NSP6, Non-Structural Protein 6; ORF3a, open reading frame 3a; NF-κB, nuclear factor-κB; NLRP3, nucleotide-binding oligomerization domain (NOD)-like receptor (NLR) family, pyrin domain-containing protein 3; ASC, apoptosis-associated speck-like protein containing a caspase recruitment domain; N, nucleocapsid; S, spike; NLRC4, NLR family, caspase activation and recruitment domain (CARD)-containing protein 4; AIM2, absent in melanoma 2; pro-IL-1β; the proform of interleukin-1β; pro-IL-18; the proform of interleukin-18; IL-1β, interleukin-1β; IL-18, interleukin-18; LDH, lactate dehydrogenase; GSDMD, gasdermin D; GSDMD-N, the N-terminal domain of gasdermin D; NSP13, Non-Structural Protein 13; NSP1, Non-Structural Protein 1; S1-RBD, receptor-binding domain in the S1 subunit; NSP5, Non-Structural Protein 5; NLRP1, NLR family, pyrin domain-containing protein 1; pro-IL-16; the proform of interleukin-16; IL-16, interleukin-16; GSDME, gasdermin E; GSDME-N, the N-terminal domain of gasdermin E; DAMPs, damage-associated molecular patterns.

Pyroptosis is a highly inflammatory form of programmed cell death that is manifested by GSDM-mediated membrane pore formation, cell swelling, cell membrane rupture, and release of cytoplasmic contents 142. The pyroptosis pathway contributes to the gathering of immune cells, thus perpetuating the inflammatory cascade. Pyroptosis is well acknowledged as a fine-tuned process in host defense system and plays an important role in resisting pathogen invasion. Nevertheless, pyroptosis could bring about devastating hyper-inflammation 143. Multiple lines of evidence support the interconnection between SARS-CoV-2 infection and host cell pyroptosis (Figure 3). As SARS-CoV-2 infection can elicit intense immune responses due to the inflammatory cytokine storm, it is reasonable to postulate that pyroptosis-mediated inflammation may be associated with the pathogenesis of COVID-19.

## SARS-CoV-2-mediated inflammasome activation

Inflammasomes are multimeric protein complexes that recognize danger signals and induce caspase-1-mediated pyroptotic cell death 144. Four key inflammasomes, namely NLRP1, NLRP3, AIM2 and NLR family, caspase activation and recruitment domain (CARD)-containing protein 4 (NLRC4), have been well characterized 145. ASC is required for the formation and function of NLRP3 and AIM2 inflammasomes. NLRP1 and NLRC4 inflammasomes can function with or independent of ASC. Monocytes possess various inflammasome-priming pathways, making them rapidly respond to extra stimuli 146. Monocytes and macrophages are sentinel cells that recognize pathogen invasion to initiate the assembly of inflammasomes, culminating in pyroptosis and inflammatory responses 147. Monocytes serve as a major driver of the deleterious inflammation and cytokine storm in COVID-19 patients 148. Through integrative analysis of single-cell RNA sequencing data of immune cells from moderate and severe COVID-19 patients, the expression levels of pyroptosis-associated markers NLRC5, caspase-1, caspase-4, GSDMD, Ninjurin 1 (NINJ1), IL-1β and IL-18 were found to be significantly higher in severe patients than moderate patients 137. Particularly, monocyte-derived macrophages showed a markedly increased expression of pyroptosis markers among all immune cells investigated. Moreover, the activation of pyroptosis-related pathways, such as NF-κB signaling and IL-1R signaling, was more pronounced in macrophages from the severe group, suggesting that monocyte-derived macrophages manifested high pyroptosis activity and might be a key player in SARS-CoV-2-assocaited cytokine storm. Pyroptosis could enhance the excessive secretion of proinflammatory cytokines and lymphopenia. Collectively, pyroptosis is tightly associated with the clinical outcome of COVID-19 patients, and may represent a therapeutic target to relieve the cytokine storm in COVID-19. These findings provided single-cell transcriptomic evidence for the hyper-activation of pyroptosis in severe COVID-19 patients. The pyroptosis state in immune cells from COVID-19 patients merits further survey.

The plasma levels of GSDMD, proinflammatory cytokines (IL-1β and IL-18) and LDH were increased in COVID-19 patients versus healthy controls 11. The expression of these pyroptosis-related proteins closely correlated with disease severity. These factors could be exploited as diagnostic biomarkers to help identify patients who may be prone to develop exuberant inflammatory complications and benefit from immunotherapy. Furthermore, canonical inflammasomes NLRP3 and AIM2, caspase-1 and GSDMD were activated in COVID-19 monocytes 11. Accordingly, SARS-CoV-2 infection could cause pyroptotic cell death in circulating monocytes. Further study showed that SARS-CoV-2 infection of monocytes was mainly mediated by uptake of antibody-opsonized virus via Fc receptors CD16 and/or CD64. SARS-CoV-2 started to replicate after it entered monocytes. Paradoxically, infectious viral particles could not be detected in infected monocyte supernatants or COVID-19 patient plasma. It was inferred that SARS-CoV-2-induced inflammatory cell death halted production of progeny viruses in infected monocytes. Instead, this form of lytic cell death resulted in systemic inflammation that propelled COVID-19 progression. Furthermore, inflammasomes were activated in lung tissue-resident macrophages from COVID-19 patients, which led to a hyper-inflammatory state of the lung tissues. Conversely, interruption of the NLRP3 inflammasome signaling mitigated chronic lung pathology, suggesting that inflammasome activation and the resultant inflammatory response served as the chief causes of lung damage 67. Repression of NLRP3 inflammasome activation also enhanced the production and release of newly generated virions by infected macrophage. Altogether, immune cells appear to fight SARS-CoV-2 infection through generation of proinflammatory cytokines and self-destruction via inflammasome-dependent pyroptosis to prevent infectious virion production. Pyroptosis in virus-infected macrophages/monocytes may act as an immune alarm to attract immune cells to infection sites, culminating in activation of innate and adaptive immune responses. This may provide a plausible explanation for high SARS-CoV-2 antibody responses in severe COVID-19 cases 149. In addition, proinflammatory factors released from pyroptotic macrophages/monocytes results in a cytokine storm. Pyroptotic myeloid cells may be a principal culprit for serious inflammatory sequela that contribute to multiorgan failure, vascular leakage and ARDS in COVID-19. The importance of macrophage/monocyte pyroptosis in COVID-19 immunopathogenesis needs to be determined. It is essential to investigate whether other types of infected immune cells act as potential sources of deleterious inflammation. NLRP3 and AIM2 inflammasomes that detected cell membrane damage and cytosolic DNA were activated in SARS-CoV-2-infected monocytes 11. It is worthwhile to ascertain whether the inflammasomes (e.g., NLRP1 and NLRP6) that recognize double-stranded RNAs (dsRNAs) are assembled. The mechanisms responsible for SARS-CoV-2-mediated inflammasome activation in monocytes necessitate additional attention.

Similarly, another study also verified that SARS-CoV-2 ignited the activation of NLRP3 inflammasome and caspase-1, enhanced the generation of cleaved GSDMD, and promoted the release of LDH and IL-1β, thereby triggering lytic cell death in human primary monocytes 136. Suppression of IL-1R engagement limited SARS-CoV-2-mediated caspase-1 activation and lytic monocyte death, hinting that inflammasome-induced IL-1β release aggrandized caspase-1 activity and pyroptosis in virus-infected monocytes. Moreover, depletion of caspase-1, NLRP3 or IL-1R hindered SARS-CoV-2-mediated upregulation of IL-1β, IL-6 and TNF-α. This result demonstrated that NLRP3 inflammasome activation augmented inflammatory responses in infected monocytes. Impediment of early steps of SARS-CoV-2 life cycle in turn prevented pyroptotic cell death in human primary monocytes 136. It is anticipated that SARS-CoV-2 infection-mediated lytic monocyte death may contribute to intense leukopenia and uncontrolled inflammation in severe COVID-19. SARS-CoV-2 NSP6 may affect intercellular potassium (K^+^) channels to activate NLRP3 inflammasome 150. Further study is still required to corroborate this hypothesis. In addition to monocytes, SARS-CoV-2 can cause pyroptosis in other cells. For instance, SARS-CoV-2 infection was shown to induce pyroptotic cell death in human microvascular endothelial cells coincident with activation of the NLRP3/caspase-1 signaling cascade and IL-1β maturation 151. *In vivo* experiment showed that SARS-CoV-2 infection could trigger the NLRP3 inflammasome signaling and pyroptosis in the lungs of the transgenic K18-human ACE2 (hACE2) mice. IL-1β interrupted lung endothelial barrier through downregulation of cAMP response element-binding protein (CREB)-mediated vascular endothelial (VE)-cadherin expression and induced gathering of neutrophils, leading to the exaggerated inflammation state 152. IL-1R antagonist anakinra diminished SARS-CoV-2-induced ARDS, lung fibrosis and mortality in mice 151. Inhibition of SARS-CoV-2-activated inflammasome/capase-1/IL-1β signaling pathway could prevent lung vascular endothelial injury, edema formation and respiratory failure. IL-1R-specific antagonists are likely to possess beneficial effects in COVID-19 treatment. Based on the above-mentioned studies, it can be concluded that SARS-CoV-2 infection has the ability to evoke diverse inflammasome signalings. However, continuous efforts should be undertaken to thoroughly understand how SARS-CoV-2 activates the canonical inflammasomes. The constant emergence of new SARS-CoV-2 variants of concern exhibiting increased infectivity emphasizes the urgent need for effective therapeutic interventions for COVID-19. The wide-scope screening of pharmaceuticals that can specifically target activated inflammasomes in COVID-19 should be a central task for future research.

## The role of SARS-CoV-2 proteins in pyroptosis induction

SARS-CoV-2 S protein is a trimeric class I fusion protein consisting of S1 and S2 subunits and plays a crucial role in SARS-CoV-2 pathogenesis 153. The implication of SARS-CoV-2 S protein in regulation of host cell pyroptosis has been well documented (Figure 3). It was reported that SARS-CoV-2 S protein motivated NLRP3 inflammasome-mediated pyroptosis in hematopoietic stem/progenitor cells (HSPCs) and endothelial progenitor cells (EPCs) 154. The secretion of LDH and IL-1β was also enhanced in these cells after exposure to S protein, while inhibition of NLRP3 inflammasome activation exerted the opposite effect. Likewise, upregulation of ACE2 and suppression of TLR4 markedly repressed pyroptotic cell death. Therefore, targeting NLRP3 might be helpful in diminishing damage of HSPCs and EPCs during COVID-19 pathogenesis. Considering the presence of several S variants, the biological functions of these variants are worthy of detailed investigations. The exact effects of SARS-CoV-2 S protein on pyroptosis remain to be validated in similar studies using either pseudotyped or appropriate life variants of the virus. ACE2 and TMPRSS2 were highly expressed in human corneal epithelial cells (HCECs), suggesting the susceptibility of HCECs to SARS-CoV-2 infection 155. SARS-CoV-2 S protein decreased the expression level of IL-6, IL-8 and TNF-α in HCECs, while increased that of GSDMD and IL-1β. As a result, SARS-CoV-2 S protein repressed host inflammatory responses and triggered pyroptosis in HCECs. Contradictorily, another study showed that SARS-CoV-2 S protein induced a robust inflammatory response in HCECs, as evidenced by elevated release of proinflammatory cytokines, including IL-1β, IL-6, IL-8 and TNF-α 156. Thus, the precise role of S protein in HCECs needs to be further examined.

Mesenchymal stem cells (MSCs) exhibit anti-inflammatory activities and can be genetically engineered to carry mediations or target genes. MSC-based treatment may be an effective, tailored cell therapy suited to COVID-19-associated ARDS and acute lung injury 157. The efficacy of MSC-based therapies in improving SARS-CoV-2-induced immunopathology was previously uncovered 158. SARS-CoV-2 S protein induced pyroptosis and inflammatory responses in bronchial epithelial cells, as evidenced by upregulation of GSDMD, the existence of multitudinous pores on the cytoplasmic membrane, activation of NLRP3 and NLRC4 inflammasomes, and massive production of IL-1β and IL-18. ACE2-overexpressing MSCs (ACE2-MSCs) could suppress inflammation and pyroptosis induced by SARS-CoV-2 S protein. Pseudoviruses expressing SARS-CoV-2 S protein could simulate the authentic virus to infect host cells without replication and had been widely exploited as an alternative strategy for studying SARS-CoV-2 pathology 159. SARS-CoV-2 pseudoviruses exacerbated lipopolysaccharide (LPS)-induced acute lung injury in mice 158. Mechanistic investigation indicated that SARS-CoV-2 pseudoviruses elevated the expression levels of core factors of the cytokine storm activated by LPS treatment, such as TNF-α, IL-6, IL-10, CCL2 and C-X-C motif chemokine 10 (CXCL10). They also potentiated LPS-induced pyroptosis in mice by enhancing the expression of NLRP3, AIM2, ASC and caspase-1. Administration of ACE2-MSCs ameliorated lung injury and aborted the cytokine storm and pyroptotic cell death in lung tissues of SARS-CoV-2 pseudovirus-infected hACE2 transgenic mouse model. Altogether, ACE2-MSCs have the potential to inhibit pyroptosis and cytokine storm stirred up by SARS-CoV-2 and thus may be effective in lessening COVID-19-induced lung injury. Notably, the immunopathogenesis of pseudoviruses expressing SARS-CoV-2 S protein cannot completely mimic the genuine virus. In other words, the therapeutic benefits of ACE2-MSCs in this COVID-19 model are probably not sound enough for clinical research. Further investigation with competent virus infection is of great significance to substantiate the effectiveness of ACE2-MSC-based therapies in treating COVID-19. Another important future goal will be to disclose the molecular mechanisms underpinning the role of ACE2-MSCs in regulating pyroptosis and cytokine storm in COVID-19.

SARS-CoV-2 S1 induced pyroptotic cell death in human visceral adipocytes through activation of the NLRP3 inflammasome signaling, induction of GSDMD cleavage and IL-1β release 160. The myokines fibronectin type III domain-containing protein 4 (FNDC4) and FNDC5 restrained SARS-CoV-2 invasion and viral S1-induced inflammatory cell death in visceral adipocytes through downregulation of the viral receptors ACE2, CD147, dipeptidyl peptidase 4 (DPP4) and neuropilin 1 (NRP1). The decreased expression of FNDC4 and FNDC5 is one possible explanation for COVID-19 deterioration in patients with obesity. In addition to pyroptosis, other inflammatory cell death modes including apoptosis and necroptosis also occurred in adipocytes treated with S1. The coordination pattern between SARS-CoV-2 S1-induced cell death pathways during COVID-19 pathogenesis could be an important research focus that needs to be addressed in future studies. The proinflammatory cytokines, including IL-1β, IL-6 and IL-18, were highly expressed in the infiltrating pulmonary macrophages of COVID-19 patients, indicating that proinflammatory macrophages were implicated in SARS-CoV-2-induced cytokine storm 26. Mechanistically, the open reading frame 3a (ORF3a) protein of SARS-CoV-2 triggered GSDMD-executed pyroptosis in the infiltrating macrophages and thus stimulated the secretion of proinflammatory mediators. Perturbation of the pyroptosis pathway may represent a promising strategy for controlling the cytokine storm in COVID-19 patients. The RBD in the S1 subunit (S1-RBD) of SARS-CoV-2 was essential in binding to ACE2 receptor 161. SARS-CoV-2 S1-RBD and ORF3a induced caspase-1-mediated pyroptosis in human bronchial epithelial cells, which was accompanied by extravasation of IL-1β and HMGB1. HMGB1 is a well-characterized DAMP protein and plays an important role in the pathogenesis of various inflammatory disorders of infectious origin 162. Inhibition of HMGB1 proinflammatory activities has been suggested as a potential therapeutic approach that needs further investigation. Consistently, glycyrrhizin, a HMGB1 inhibitor, suppressed caspase-1 activation and protected human bronchial epithelial cells from SARS-CoV-2-induced pyroptotic cell death 161. As expected, glycyrrhizin diminished the release of proinflammatory cytokines from macrophages cultivated in conditioned media from lung cells transfected with SARS-CoV-2 S1-RBD and ORF3a. Nevertheless, the therapeutic efficacy of glycyrrhizin in blunting exuberant inflammation remains unclear and should be the focus of upcoming studies. SARS-CoV-2 S1-RBD and ORF3a could cause lung pathology through heightened death and secretion of IL-1β and HMGB1. By contrast, it appeared that malignant lung, stomach and kidney cell lines were insusceptible to these viral proteins-induced pyroptosis. Tissue type-specific distinctions in responsiveness to SARS-CoV-2 proteins underscore the sophisticated interaction between the virus and host tissues leading to excessive inflammation. This interaction plays a central role in viral infection and pathogenesis, making it be an interesting field of future study.

Interruption of the autophagic flux is capable of inducing NLRP3 inflammasome activation 163. SARS-CoV-2 NSP6 could instigate pyroptosis in lung epithelial cells by inducing capase-1 cleavage and GSDMD activation, and promoting IL-1β and IL-18 maturation 164. In terms of mechanism, NSP6 impaired lysosome acidification and restricted the lysosome-autophagosome system in lung epithelial cells by interacting with the lysosomal proton pump component, ATPase H^+^ Transporting Accessory Protein 1 (ATP6AP1). This event resulted in termination of the autophagic flux and induction of NLRP3 inflammasome-dependent pyroptosis. Conversely, pharmacological restoration of the autophagic flux abrogated NSP6-induced pyroptosis. Downregulation of caspase-1, NLRP3, ASC or caspase-1 could not completely dampen NSP-6-induced pyroptosis, implying that other pyroptosis-related pathways also operated. Autophagy serves as a critical host defense mechanism to resist microbial invasion. It not only has the ability to degrade invading pathogens, but also favors antigen presentation and augments adaptive immune responses 163. Nevertheless, the virus has evolved diverse strategies to manipulate this lysosome-dependent degradation process for its infection and propagation 165. A recent report manifested that SARS-CoV-2 ORF3a protein acted to suppress autophagosome-lysosome fusion by targeting vacuolar protein sorting 39 (VPS39), a component of the homotypic fusion and vacuole protein sorting complex 166. It is unclear whether other virulence factors of SARS-CoV-2 can interfere with the same process to promote COVID-19 pathogenesis. Emerging evidence has suggested that destruction of lysosomal function or repression of autophagic degradation can trigger different forms of cell death including apoptosis and necroptosis 167. It is possible that other cell death modes mediate the cytotoxic actions of NSP6 and ORF3a. Additional work is required to delve into the regulatory actions and molecular mechanisms of these viral proteins in cell death pathways.

## Inhibition of pyroptosis by SARS-CoV-2

SARS-CoV-2 has evolved to counteract the pyroptosis pathway in infected cells. It was reported that SARS-CoV-2 N protein could directly bind to NLRP3 and facilitated its interaction with ASC 168. Thus, N protein fostered the assembly and activation of NLRP3 inflammasome, promoting the production of various inflammatory factors (e.g., IL-1β, IL-6, CCL-2 and CXCL10). N protein worsened lung injury and promoted death in acute inflammation mouse models by promoting NLRP3 inflammasome activation. Conversely, inhibition of NLRP3 and caspase-1 could counteract N protein-induced lung damage and inflammatory responses. It appears that the binding of N protein to NLRP3 is critical for priming NLRP3 inflammasome. Intriguingly, N protein has no influence on the production of IL-18, which is an effector molecule downstream of NLRP3 inflammasome. The effects of N protein on the NLRP3 inflammasome signaling are worthy of in-depth research. Suppression of NLRP3 inflammasome activation may alleviate SARS-CoV-2-induced cytokine storm and lung injury. The therapeutic benefits of NLRP3 inflammasome inhibitors warrant additional evaluation. Although viral N protein regulates the activity of NLRP3 inflammasome in SARS-CoV-2-infected immune cells, it impedes the downstream pathway of pyroptosis. In line with the aforementioned study, Ma et al. 9 revealed that SARS-CoV-2 infection stirred up the NLRP3 inflammasome signaling in monocytes. Intriguingly, IL-1β release and pyroptosis were inhibited in virus-infected monocytes. In terms of mechanism, N protein of SARS-CoV-2 bound the C-terminal domain and the linker region of GSDMD and protected GSDMD from caspase-1-mediated cleavage. Moreover, there were multiple interfaces between viral N protein and GSDMD, alluding to a higher tertiary structure employed by N protein to capture GSDMD. SARS-CoV-2 nucleocapsid is released into host cytosol once the virus enters the host cell. The binding of SARS-CoV-2 N protein to host factors may be beneficial for long asymptomatic viral infection and the inhibition of host immune responses immediately post its invasion into host cells. Host cells may reserve abundant proinflammatory mediators in the cytosol owing to N protein-mediated GSDMD inhibition. The lytic infection of SARS-CoV-2 is likely to abolish this inhibition, which actuates transient extravasation of massive inflammatory factors and eventually leads to severe symptoms in COVID-19 patients. However, GSDMD downregulation possibly induces other modes of cell death, which may result in the reduction of non-classical monocytes. The role of GSDMD inhibition in COVID-19 immunopathology remains to be unraveled. In short, N protein-induced NLRP3 inflammasome activation contributes to SARS-CoV-2 immunopathology, while the cease of GSDMD-executed pyroptosis by N protein facilitates viral persistent infection. These findings will open up new horizons in developing alternative therapeutic measures against COVID-19 in the future. Viral proteins may affect different molecules in the pyroptosis pathway, which could be the subject of future studies. The interplay between pyroptosis blockade by N protein and initiation by viral factors warrants detailed investigation.

NLRP1 was shown to act as a sensor of SARS-CoV-2 in human lung epithelial cells 169. SARS-CoV-2 3CL protease NSP5 mediated NLRP1 cleavage at the Q333 site and favored its activation, which drove NLRP1 inflammasome assembly, while it interrupted the NLRP1 inflammasome signaling by inactivating the pyroptosis executioner GSDMD (Figure 3). Nevertheless, SARS-CoV-2-infected lung epithelial cells underwent lytic cell death, even in the absence of activated GSDMD, which alluded to the engagement of other pyroptosis effectors. It turned out that the caspase-3/GSDME pathway functioned as the sentinel that mediated lung epithelial cell pyroptosis in the process of SARS-CoV-2 infection if the key pyroptosis executioner GSDMD was inactivated. Consequently, NLRP1-dependent pyroptosis restricted the production of infectious virus and promoted the release of alarmins and DAMPs. In addition, the plasma levels of pyroptosis-associated markers including caspase-3, GSDME and IL-18 intimately correlated with disease severity in COVID-19 169. It is still equivocal how viral proteases specifically target NLRP1 and GSDMD. The ability of viral proteases to modulate the inflammasome pathway may be beneficial for the establishment of a favorable replicative niche by viruses in infected cells. The NLRP3 inflammasome pathway in myeloid cells partially accounts for the cytokine storm in COVID-19 patients 25. The effect of NLRP1 inflammasome activation on NLRP3-mediated response in patient myeloid cells is another key question that ought to be addressed in future studies. It is tempting to speculate that SARS-CoV-2-derived proteins counteract GSDMD-executed pyroptosis to promote robust viral replication, while the host has to adopt alternative pyroptosis pathways, which may lead to delayed host immune defense and exaggerated inflammation. Accordingly, NLRP1 inflammasome-dependent pyroptosis could exert both beneficial and deleterious effects on viral infection and pathogenesis. Additional work is necessary to unravel the implication of NLRP1 inflammasome in SARS-CoV-2-induced cytokine storm and organ dysfunction. Altogether, these findings indicated that NLRP1 behaved as an innate immune sensor of SARS-CoV-2 and revealed the virulence mechanisms through which this virus resisted host intrinsic immune response.

NSP1 and NSP13 were reported to antagonize the NLRP3/caspase-1 pathway-mediated IL-1β maturation 170. Mechanistically, NSP1 stopped the translational machinery in host cells by binding to the 40S ribosome subunit, which eventually led to caspase-1 inhibition. By contrast, NSP13 interfered with NLRP3 inflammasome-mediated cleavage of caspase-1. It was proposed that NSP13 helicase activity was involved in caspase-1 inhibition. Much work is required to understand the mechanism by which NSP13 regulates capase-1 activity. Moreover, it remains elusive whether other NSPs of SARS-CoV-2 can coordinate the pyroptosis pathway and participate in COVID-19 pathogenesis via the same mechanism. On the other side, SARS-CoV-2 may affect inflammasome activation via diverse mechanisms. To persist in host cells, viruses must escape host immune attack. Suppression of inflammasome activation can be either protective or destructive relying on the stage of viral infection. NSP1 and NSP13 are synthesized during the early infection stage and exert an inhibitory effect on NLRP3 inflammasome activation. SARS-CoV-2 may dampen host inflammatory responses through production of NSP1 and NSP13, which facilitates viral replication and assembly. Late viral products (e.g., S and ORF3a) function to motivate inflammasome-mediated lytic cell death, enabling viral spread and pathogenesis. However, the strategies exploited by SARS-CoV-2 to control inflammasome activity remain largely fragmented and await thorough illumination. The crosstalk between pyroptosis induction and inhibition by virulence factors of SARS-CoV-2 deserves in-depth investigation. Concerted efforts are required to identify the key parameters determining which pyroptosis pattern takes precedence over others under distinct circumstances.

## Roles of pyroptotic cell death in COVID-19 immunopathogenesis

Compelling evidence has identified that the pyroptosis pathway plays a momentous role in COVID-19 immunopathogenesis. The ACE2 receptor is present on various types of cells, such as respiratory epithelial cells and tissue-resident macrophages, which allows SARS-CoV-2 to infect these cells 171. Particularly, lung epithelial cells are easily to suffer from SARS-CoV-2 invasion due to their large region of exposure to external milieu. Initiation of the inflammasome signaling in lung epithelial cells was reported to have a relationship with exuberant tissue inflammation and viral pathogenesis 172. However, it remains to substantiate whether pyroptosis in lung epithelial cells acts as a contributor to severe pneumonia in COVID-19.

Pyroptotic macrophages that have phagocytosed viruses can cause the liberation of multitudinous alarmins, such as cytokines, chemokines, LDH and reactive oxygen species (ROS), eliciting an expeditious reaction from proximate immune cells and a pyroptosis chain reaction. Pyroptosis could permit viral components (e.g., RNA and antigens) to be released in the circulation, which possibly results in the genesis of immune complex and accumulation of immune cells in target tissues to actuate critical inflammatory cascade. Pyroptosis in alveolar macrophage causes acute lung injury by fostering infiltration of neutrophils and monocyte-derived macrophages into the lung tissues and increasing alveolar contents of proinflammatory cytokines (e.g., IL-1β, IL-6 and TNF-α) 173. These events contribute to excessive release of inflammatory mediators, more death of neighboring cells and further innate immune cell attraction, all of which dramatically fuel the pathology of pneumonia in COVID-19 patients. In addition, pyroptotic immune cell-secreted proinflammatory cytokines IL-1β and IL-6, which were present at high levels in COVID-19 patients, might aggrandize lymphopenia via direct killing of lymphocytes, contributing to the dysfunction of adaptive immunity in COVID-19 174. As a result, the host may fail to curb the inflammation that is potentiated by proinflammatory factors originated from the cell death, which leads to a self-destructive shutdown of the immune system, also known as acute virus-induced immune deficiency (AVID). Thrombotic complications including pulmonary embolism caused by aberrant blood coagulation are linked with poor clinical outcomes in COVID-19 patients 175. TF acts as a key inducer of coagulation cascades. Reportedly, TF released by pyroptotic macrophages could induce blood coagulation and substantial thrombosis in tissues 109. It is possible that pyroptotic macrophage-derived TF leads to organ damage in COVID-19 patients. The repression of inflammasome-dependent pyroptosis in macrophages may reduce system blood clotting by restricting TF dissemination in the circulation. However, this speculation needs further corroboration.

Collectively, hyper-activation of the pyroptosis pathways leads to the cytokine storm and severe injury to host. Pyroptosis has thus been the focus of extensive study. Studying pyroptotic cell death during SARS-CoV-2 infection could provide a better comprehension of COVID-19 pathophysiology and will contribute to the development of better therapeutic approaches that can alleviate the damage caused by SARS-CoV-2-associated immune responses. Significant reduction of certain types of immune cells, including lymphocytes and monocytes, is the major driver of COVID-19 severity. The accurate mechanisms underlying lymphopenia and monocytopenia are yet to be elucidated. Currently, most COVID-19 studies mainly rely on *in vitro* studies or correlative datasets derived from patient samples. Moreover, it is obscure whether pyroptosis affects COVID-19 pathogenesis independent of cytokine release. Therefore, continued studies are needed to determine the role of pyroptosis in COVID-19 immunopathology. The engagement of other cell death pathways such as apoptosis and necroptosis has also been proposed. Different cell death modes may act synergistically in COVID-19. Whether pharmaceutical targeting of the pyroptosis pathway is sufficient to achieve successful management of COVID-19 is an important future question to pursue.

## Potential therapeutic implication of the pyroptosis pathway in COVID-19

NLRP3 inflammasome activation is involved in the development of various disorders, including cardiovascular diseases, chronic obstructive pulmonary disease, hypertension and type 2 diabetes 176. Underlying comorbidities are considered as a primary reason for disease severity and death in COVID-19 patients 177. Pharmacological blockade of NLRP3 inflammasome may ameliorate underlying comorbidities associated with COVID-19, thus improving COVID-19 prognosis and attenuating the risk of death. The appraisal of clinical efficacy of several compounds with modulatory effects on NLRP3 inflammasome activity in COVID-19 treatment is currently being addressed. The tryptophan analogue tranilast is a representative example, which functions as a NLRP3 inhibitor and has been approved for the clinical treatment of allergic and inflammatory disorders 178. It holds the potential as an attractive therapeutic agent for the management of COVID-19 patients with comorbidities. At present, the therapeutic potency of tranilast is being evaluated in a randomized control trial in COVID-19 patients. Colchicine, an alkaloid compound separated from plant Colchicum, has been applied in treatment of inflammatory diseases 179. It can suppress the activity of NLRP3 inflammasome to limit the actions of inflammatory cells and to prevent discharge of proinflammatory cytokines. Although colchicine may bring about several side effects, such as diarrhea, nausea and vomit, it still presents a prospective candidate for reducing SARS-CoV-2-induced hyper-inflammation. In randomized controlled trials, colchicine treatment significantly reduced mortality in COVID-19 patients 180. Nevertheless, the benefits of colchicine deserve thorough evaluation in COVID-19 clinical trials.

Suppression of downstream pathways of inflammasome activation also represents an appealing therapeutic option. Caspase-1 was found to be upregulated in CD4^+^ T cells from COVID-19 patients versus healthy controls 181. *In vitro* experimental studies showed that pan-caspase inhibitor emricasan (EMR) could reduce active caspase-1 in CD4^+^ T cells from COVID-19 patients. A phase I clinical trial is under way to determine the safety and tolerability of EMR in COVID-19 patients. Inflammasome activation serves as an important innate immune defense against invading pathogens. Nevertheless, inflammasome-mediated pyroptosis in immune cells causes massive release of inflammatory and danger signals, which could be detrimental to the host. The exuberant caspase response might cause immune-relevant pathological process in COVID-19. Blockade of the pyroptosis pathway in immune cells by using caspase inhibitors would represent an effective treatment approach. However, it remains to verify whether pan-caspase inhibition can suppress inflammasome activation and prevent disease progression in COVID-19 patients. Disulfiram is an approved drug used to treat alcohol addiction 182. Disulfiram suppressed GSDMD-mediated pore formation, hence hindering IL-1β liberation and pyroptosis. Disulfiram was likely to antagonize intense inflammation during COVID-19 pathogenesis. A retrospective cohort study demonstrated that disulfiram was associated with decreased incidence and severity of COVID-19 183. Despite these encouraging results, clinical trials are still necessary to carefully determine the therapeutic efficacy of disulfiram in COVID-19 patients.

Pyroptosis acts as a critical mechanism of cytokine release resulting from cell membrane disruption 17. Massive production of proinflammatory cytokines causes widespread tissue insult leading to multiorgan dysfunction and death in COVID-19 patients 184. Targeting cytokines holds the potential to improve clinical outcomes in COVID-19 patients. Anakinra and canakinumab are representative examples. Anakinra is an IL-1R antagonist used to treat rheumatoid arthritis and autoinflammatory diseases 185. Canakinumab is a human monoclonal antibody targeting IL-1β that was initially developed to treat autoinflammatory syndromes 186. As is known, IL-1β plays an important role in inducing cytokine storm in COVID-19 patients. Previous studies demonstrated that treatment with anakinra or canakinumab markedly reduced the utilization of invasive mechanical ventilation and contributed to favorable prognosis without severe side effects in COVID-19 patients 187, 188. Clinical trials are being performed to assess the therapeutic effectiveness and safety of anakinra and canakinumab in COVID-19 patients. Another cytokine-targeted strategy involves IL-6 blockade with monoclonal antibodies. The expression level of IL-6 shows an apparent upward trend in COVID-19 patients and is tightly associated with ARDS severity and poor prognosis. IL-6 may be a prospective target cytokine to treat COVID-19-associated ARDS. Tocilizumab, a recombinant humanized anti-IL-6R monoclonal antibody, has been approved for the treatment of cytokine release syndrome and rheumatologic disorders 189. Recent studies have proven that critically ill COVID-19 patients could benefit from tocilizumab treatment, opening up new avenues for therapeutic intervention against COVID-19 190. Large, randomized control trials are necessary to determine the effectiveness of IL-6 inhibition among COVID-19 patients. Curcumin, as the medicinal component of turmeric, exhibits diverse pharmacological benefits such as antioxidant and anti-inflammatory activities 191. Curcumin may have a role in treating inflammation-related diseases. A randomized, double-blind, placebo-controlled study revealed that nano-curcumin treatment reduced the serum levels of IL-1β, IL-6 and TNF-α in COVID-19 patients 191. Importantly, nano-curcumin evidently ameliorated the clinical manifestations in COVID-19 patients, such as cough, dyspnea and fever. Nano-curcumin may be a novel therapeutic agent for COVID-19. The pharmacological mechanisms, therapeutic potency and potential side effects of nano-curcumin in COVID-19 patients need to be systematically explored.

Hyper-inflammation and lung dysfunction are key pathophysiological hallmarks of COVID-19. The equilibrium between viral pathogenicity and host immunity has a significant impact on COVID-19 severity. Inflammasome assembly is provoked in response to a variety of PAMPs that emerge during pathogen infections, followed up by the production of proinflammatory cytokines and chemokines including IL-1β, IL-6, IL-18 and TNF-α. Generally, a sound immune system can efficiently eradicate the infection, whereas a compromised immune system causes excessive accumulation of active immune cells in the lungs, contributing to a cytokine storm and multiorgan failure via circulation. Harnessing the extravagant inflammation may be a potential therapeutic strategy for COVID-19 treatment. Pyroptosis-associated inflammation has a close relationship with COVID-19 immunopathogenesis. A growing body of evidence has manifested the therapeutic potential of pyroptosis regulators in the management of COVID-19. Further clinical trials aimed at controlling immune-related damages in COVID-19 are under way. It is of great necessity to corroborate the therapeutic benefits, determine the optimal dose, route, frequency and timing of administration, and investigate the safety, tolerability and pharmacokinetics of drug candidates, all of which may make it feasible for pyroptosis-targeting therapies to cope with COVID-19.

## Conclusions and perspectives

Pyroptosis is a pivotal cell response to microbial infections and non-infectious stimuli, which has been implicated in the pathogenic mechanisms of various diseases. The crosstalk between SARS-CoV-2 and the pyroptosis pathway has been preliminarily unveiled. SARS-CoV-2 infection can induce pyroptosis in host cells, and the propagation and spread of this virus are prevented owing to the removal of infected cells via pyroptosis. However, SARS-CoV-2 infection causes uncontrolled cell death, leading to extensive cell damage and unfavorable immune responses. Meanwhile, the virus can delicately coordinate this inflammatory cell death pathway to drive its replication and pathogenesis. The mechanisms exploited by the virus to regulate pyroptosis are worthy of further investigations. More studies are required to figure out the contribution of pyroptosis to SARS-CoV-2 infection and COVID-19 development. The cytokine storm is a main promotor of COVID19 progression, while the production and release of cytokines are closely associated with pyroptosis. It is arduous to illuminate the separate role of pyroptosis in COVID-19. Furthermore, the causal relationship between pyroptosis and cytokine release should also be determined. There are few studies using preclinical animal models (e.g., hACE2 transgenic mice) to elucidate the role of pyroptosis during SARS-CoV-2 infection. Most studies have revealed the effects of canonical inflammasome pathways on SARS-CoV-2 pathogenicity, yet it is unclear whether other pyroptosis pathways such as apoptotic caspase-mediated pathway and non-canonical inflammasome pathways are engaged in the pathological process of SARS-CoV-2. In-depth investigations of pyroptosis and its implication in COVID-19 immunopathology would provide new insights into the pathogenic mechanisms of SARS-CoV-2 and help to design efficacious therapeutic approaches for COVID-19 treatment.

Pharmacological regulation of the pyroptosis pathway could be a promising intervention to combat COVID-19. Importantly, curtailing this cell death process may lessen the effect of counterproductive inflammatory factors. At present, many therapeutic measures targeting inflammasomes, caspases, GSDMD or cytokines are entering clinical trials to evaluate efficacy in mitigating COVID-19. Considering the double-edged roles of pyroptosis in SARS-CoV-2 pathogenesis, it is indeed essential to decide whether and when this cell death process should be induced or impeded. Several key components of the pyroptosis pathway, such as inflammasomes and caspases, function to regulate cytokine maturation and release, rendering them to be ideal targets for COVID-19 therapy. Overall, the clinical benefits of therapeutic manipulation of the pyroptosis pathway are multifaceted and the screen for available or new targeting agents may represent a particularly worthwhile effort. However, the safety and effectiveness of such agents must be carefully evaluated through clinical trials. In addition, the specific mechanisms responsible for their therapeutic activities also need further research before clinical translation.
